# Association between serum lipid profile and liver fibrosis in patients infected with *Schistosoma japonicum*

**DOI:** 10.1186/s13071-022-05359-8

**Published:** 2022-07-29

**Authors:** Yang Liu, PengPeng Zhang, JunHui Li, Hao Li, Chen Zhou, Yu Zhang, YingZi Ming

**Affiliations:** 1 Transplantation Center, Third Xiangya Hospital, Central South University, Changsha, China; 2 Engineering and Technology Research Center for Transplantation Medicine, National Health Commission, Changsha, 410013 China

**Keywords:** Lipid, Liver fibrosis, Schistosomiasis, High-density lipoprotein, Low-density lipoprotein, Hemoglobin

## Abstract

**Background:**

Liver fibrosis is thought to have a close relationship with lipid profile. The possible association between lipids and liver fibrosis of different etiologies has been widely explored. However, the association between lipids and liver fibrosis in patients infected with *Schistosoma japonicum* remains unclear. In the present study we undertook a preliminary exploration of the association between lipid profile and liver fibrosis, and developed a new predictive index for liver fibrosis in *S. japonicum*-infected patients.

**Methods:**

A total of 1503 patients diagnosed with *S. japonicum* at Xiangyue Hospital, China were enrolled in this retrospective study. The patients were divided into two groups, i.e., those with and those without liver fibrosis, by two experienced schistosomiasis specialists, according to the results of liver ultrasound examination. Demographic, clinical, and laboratory data were collected. Multivariable logistic models were used to estimate the independent associations between lipid profile and liver fibrosis. Receiver operating characteristic (ROC) curves were used to assess the discriminative ability of the new index in predicting liver fibrosis in patients with schistosomiasis.

**Results:**

Logistic regression analysis showed that high-density lipoprotein (HDL) [adjusted odds ratio (aOR), 95% confidence interval (CI) 7.334, 5.051–10.649; *P* < 0.001], low-density lipoprotein (LDL) (aOR, 95% CI 0.434, 0.370–0.509; *P* < 0.001), hemoglobin (HB) (aOR, 95% CI 0.979, 0.971–0.987; *P* < 0.001) and platelets (PLT) (aOR, 95% CI 0.996, 0.994–0.999; *P* < 0.001) were independently associated with liver fibrosis in patients with schistosomiasis. ROC analysis indicated that the combination of HDL, LDL and HB levels [(HDL × 100)/(LDL × HB)] had a higher area under the ROC curve (AUC = 0.773), and thus may better predict liver fibrosis than the aspartate transaminase-to-platelet ratio index (AUC = 0.608) and fibrosis index based on four factors (AUC = 0.624).

**Conclusions:**

To the best of our knowledge, this is the first study to report that HDL, LDL, HB and PLT levels are independently associated with liver fibrosis in patients with schistosomiasis. (HDL × 100)/(LDL × HB) outperformed the aspartate transaminase-to-platelet ratio index and fibrosis index based on four factors in terms of ROC, and thus could be a new predictive index for liver fibrosis. These findings may help clinicians to more easily and effectively diagnose liver fibrosis in patients with schistosomiasis.

**Graphical abstract:**

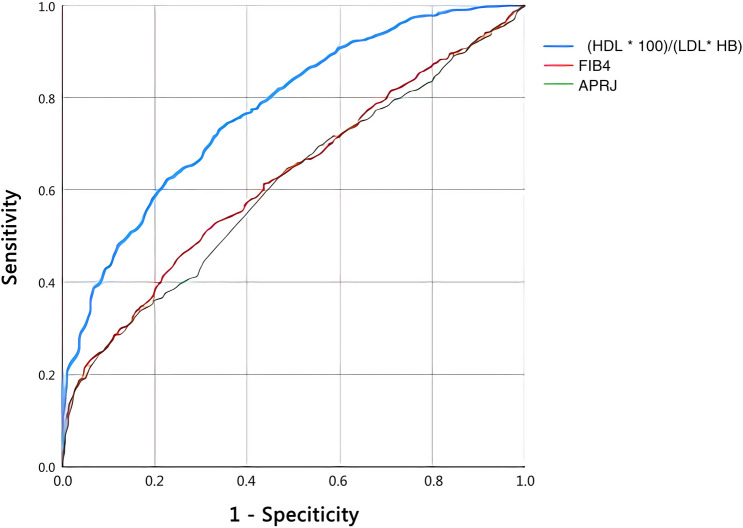

## Background

Schistosomiasis is a major public health concern worldwide, and presently affects more than 200 million people in approximately 78 countries [1]. Different *Schistosoma* species can infect humans, but only *Schistosoma japonicum* is prevalent in China [2]. Although the prevalence of *S. japonicum* continues to decline [3–5], this parasite still poses a considerable threat to humans. Previous studies have shown that liver fibrosis can continue to develop even when patients with schistosomiasis are cured by praziquantel within a reasonable period of time [6, 7]. However, liver fibrosis caused by the granulomatous response associated with the parasite’s eggs is the most serious pathological outcome and the leading cause of death in patients with schistosomiasis [2, 8, 9]. Therefore, liver fibrosis induced by *S. japonicum* infection is an increasingly important area of schistosomiasis research. It is important to note that the rapid and effective identification of liver fibrosis at an early stage is critical to the prognosis of schistosomiasis caused by *S. japonicum*.

Schistosomiasis-driven liver fibrosis is a very complex process involving host-parasite interactions. Liver fibrosis is driven by the massive deposition of *Schistosoma* eggs in the liver [10]. However, the progression of liver fibrosis is not consistent between individuals with the same egg burden [11]. A growing number of studies have suggested that specific host factors may influence the progression of liver fibrosis [11–13]. Emerging evidence suggests that lipid profile may play an important role in the development of liver fibrosis [14, 15]. Given that the liver plays a key role in lipid metabolism, the possible association between liver disease and lipid profile has always been a research hotspot.

The relationship between lipids and liver fibrosis has been explored in several studies. A cross-sectional study conducted in Spain reported that reduced high-density lipoprotein (HDL) and elevated triglycerides (TGs) were associated with significant fibrosis in chronic hepatitis B virus inactive carriers [16]. One study performed at the American University of Beirut Medical Center showed that lower low-density lipoprotein (LDL) is a predictor of severe liver fibrosis in diabetic patients with nonalcoholic fatty liver disease [17]. Additionally, a study on infected animals in China suggested that lipids may affect the development of liver fibrosis caused by *S. japonicum* infection by regulating the inflammatory microenvironment that is closely related to the scarring [18]. Despite a substantial body of research, the correlation between lipid profile and liver fibrosis remains unclear, especially with respect to *S. japonicum*. Therefore, we aimed to investigate the relationship between lipid profile and liver fibrosis, and to develop a predictive index for the presence or absence of liver fibrosis in *S. japonicum*-infected patients, which could enhance diagnostic efficiency.

## Methods

### Study design and population

A medical record review was conducted from January 2019 to June 2021 at Xiangyue Hospital, Yueyang City, Hunan Province, China. Yueyang City is located near to Dongting Lake, a flood basin of the middle and lower reaches of the Yangtze River, and its ecology and environmental factors are conducive to the reproduction of the intermediate host of *S. japonicum*, the snail *Oncomelania hupensis*. As a result, this area has historically been a high risk area for schistosomiasis.

Patients diagnosed with schistosomiasis were included in the study. All the patients underwent blood tests and ultrasound evaluation at admission. Patients infected with hepatitis B virus (hepatitis B surface antigen seropositive), hepatitis C virus (HCV antibody seropositive), or human immunodeficiency virus (HIV antibody seropositive), or who had alcoholic and non-alcoholic fatty liver disease (ultrasound scan; alcohol consumption above 30 g daily), decompensated liver disease or liver cancer (ultrasound and liver function tests), or had undergone organ transplantation (self-reported), were excluded.

Ethics approval was obtained from the Institutional Review Board (IRB) of the Third Xiangya Hospital, Central South University. According to IRB requirements for retrospective studies, consent forms were not needed for the review of patients’ medical records and data collection.

### Diagnosis of *S. japonicum* infection and liver fibrosis

*Schistosoma japonicum* infection was defined, in accordance with Zhou et al. [19], as follows: a history of living in a schistosomiasis-endemic area, contact with infested water, specific *Schistosoma* serology testing, color ultrasound, and microscopic examination of excreta (stool, urine). Visualization of parasite eggs in the stool or urine, or positive *Schistosoma* serology, were considered evidence of *S. japonicum* infection.

Liver fibrosis was determined by ultrasound in accordance with the World Health Organization standard for *S. japonicum* infection [20, 21]. Two experienced schistosomiasis specialists divided the patients into two groups based on the ultrasound findings: the fibrosis group (with mesh-like changes and an uneven hepatic echotexture), and the no-fibrosis group (without mesh-like changes and a smooth and uniform hepatic echotexture).

### Clinical evaluation and laboratory tests

The following demographic data were collected: age and gender. Anthropometric measurements including body weight and height were performed by trained nurses. The body mass index (BMI) was calculated by dividing weight (kilograms) by height squared (square meters).

Venous blood samples were collected in the morning following an 8- to 12-h overnight fasting period. Alanine aminotransferase (ALT), aspartate aminotransferase (AST), plasma fasting blood glucose (FBG), total cholesterol (TC), TG, HDL and LDL were measured using an automatic biochemical analyzer (Beckman). White blood cell count (WBC), red blood cell count (RBC), hemoglobin (HB), and platelet count (PLT) were measured using an automatic hematology analyzer (XE-5000, Sysmex).

### Statistical analysis

Statistical analysis was performed using SPSS version 26 (SPSS, Chicago, IL). Continuous variables were expressed as the mean ± SD. Student’s* t*-test or Mann–Whitney* U*-test was used to evaluate differences in general characteristics and laboratory test results between participants with and without liver fibrosis. Categorical variables were expressed as counts and proportions, and evaluated using the Chi-squared test or Fisher’s exact test. Univariate and multivariate logistic regression analysis (Forward: LR) were used to investigate the factors associated with liver fibrosis in *S. japonicum* patients. Variables with statistical significance in the univariate analysis were entered into multivariate logistic regression analysis. Spearman correlation analysis was used to determine the correlation between the parameters and liver fibrosis. A new predictive index was generated by modeling the values of the independent variables. The diagnostic values of parameters were assessed by calculating the area under the receiver operating characteristic (ROC) curves (AUC). Cut-off points were selected according to the best Youden index. The diagnostic accuracy was calculated using sensitivity, specificity, positive predictive value (PPV), and negative predictive value (NPV). A *P*-value < 0.05 (two-tailed) was considered statistically significant.

## Results

### General characteristics of the study population

Our study included 1503 participants diagnosed with *S. japonicum*, of which 764 (50.8%) had liver fibrosis. The average age was 60.59 ± 13.03 years. The majority (67.1%) of the participants were male, and the mean BMI was 23.86 ± 3.55. Clinical characteristics and laboratory parameters of patients with and without liver fibrosis are shown in Table 1. Compared to the patients without liver fibrosis, those with liver fibrosis were older, had a lower BMI, and a higher proportion were female. HDL and AST levels were significantly higher among individuals with than without liver fibrosis, while levels of TG, TC, LDL, FBG, WBC, RBC, HB, and PLT were lower.

**Table 1:** Demographic data and laboratory test results of *Schistosoma japonicum*-infected patients with or without liver fibrosis

Variables	Total (*n* = 1503)	With liver fibrosis (*n* = 764)	Without liver fibrosis (*n* = 739)	*P*
Age (years)	60.59 ± 13.03	62.37 ± 13.22	58.76 ± 12.58	< 0.001
Males (*n*, %)	1009 (67.1%)	474 (47%)	535 (53%)	< 0.001
BMI (kg/m^2^)	23.86 ± 3.55	23.22 ± 3.56	24.53 ± 3.42	< 0.001
TG (mmol/L)	1.38 ± 0.93	1.32 ± 0.90	1.64 ± 0.94	< 0.001
TC (mmol/L)	4.80 ± 0.96	4.73 ± 0.97	4.86 ± 0.95	0.010
HDL (mmol/L)	1.42 ± 0.39	1.55 ± 0.41	1.29 ± 0.31	< 0.001
LDL (mmol/L)	3.33 ± 0.86	3.04 ± 0.78	3.63 ± 0.84	< 0.001
FBG (mmol/L)	5.61 ± 1.43	5.53 ± 1.27	5.71 ± 1.57	0.014
WBC (× 10^9^/L)	5.39 ± 1.46	5.22 ± 1.56	5.56 ± 1.32	< 0.001
RBC (× 10^12^/L)	4.35 ± 0.57	4.20 ± 0.64	4.50 ± 0.43	< 0.001
HB (g/L)	133.61 ± 16.93	129.07 ± 18.70	138.31 ± 13.35	< 0.001
PLT (× 10^9^/L)	173.26 ± 55.96	163.51 ± 62.15	183.33 ± 46.69	< 0.001
ALT (IU/L)	28.17 ± 8.50	28.23 ± 9.15	28.11 ± 7.77	0.796
AST (IU/L)	29.02 ± 9.63	29.70 ± 12.38	28.31 ± 5.42	0.005

*BMI* Body mass index, *TG* triglycerides, *TC* total cholesterol, *HDL* high-density lipoprotein, *LDL* low-density lipoprotein, *FBG* fasting blood glucose, *WBC* white blood cell count, *RBC* red blood cell count, *HB* hemoglobin, *PLT* platelets, *ALT* alanine aminotransferase, *AST* aspartate aminotransferase

### Factors associated with liver fibrosis in patients infected with *S. japonicum*

As shown in Table 2, univariate logistic regression analysis revealed that age, gender, BMI, TG, TC, HDL, LDL, FBG, WBC, RBC, HB, PLT and AST levels were associated with liver fibrosis in patients infected with *S. japonicum*. Multivariate logistic regression analysis showed that elevated HDL [adjusted odds ratio (aOR), 95% confidence interval (CI) 7.334, 5.051–10.649; *P* < 0.001] levels were independently associated with a significantly increased risk of liver fibrosis. In addition, LDL (aOR, 95% CI 0.434, 0.370–0.509; *P* < 0.001), HB (aOR, 95% CI 0.979, 0.971–0.987; *P* < 0.001), and PLT (aOR, 95% CI = 0.996, 0.994–0.999; *P* < 0.001) levels were inversely associated with liver fibrosis in patients infected with *S. japonicum* (Table 2).

**Table 2:** Logistic regression analysis of factors associated with liver fibrosis in patients infected with *Schistosoma japonicum*

Variables	OR (95% CI)	*P*
Unadjusted		
Age (years)	1.022 (1.014–1.030)	< 0.001
Male	1.605 (1.291–1.995)	< 0.001
BMI (kg/m^2^)	0.898 (0.871–0.925)	< 0.001
TG (mmol/L)	0.650 (0.569–0.742)	< 0.001
TC (mmol/L)	0.871 (0.783–0.968)	0.011
HDL (mmol/L)	8.190 (5.183–11.54)	< 0.001
LDL (mmol/L)	0.394 (0.340–0.456)	< 0.001
FBG (mmol/L)	0.912 (0.846–0.983)	0.016
WBC (× 10^9^/L)	0.850 (0.790–0.913)	< 0.001
RBC (× 10^12^/L)	0.320 (0.254–0.401)	< 0.001
HB (g/L)	0.963 (0.955–0.970)	< 0.001
PLT (× 10^9^/L)	0.993 (0.991–0.995)	< 0.001
ALT (IU/L)	1.002 (0.990–1.014)	0.796
AST (IU/L)	1.025 (1.006–1.044)	0.008
Adjusted		
HDL (mmol/L)	7.334 (5.051–10.649)	< 0.001
LDL (mmol/L)	0.434 (0.370–0.509)	< 0.001
HB (g/L)	0.979 (0.971–0.987)	< 0.001
PLT (× 10^9^/L)	0.996 (0.994–0.999)	< 0.001

*OR* Odds ratio, *CI* confidence interval; for other abbreviations, see Table 1

### Spearman correlation between serum markers and liver fibrosis

Spearman correlation coefficients and two-tailed significances estimated by bivariate analysis between the parameters and liver fibrosis are shown in Table 3. LDL (*r* = − 0.318, *P* < 0.001), HB (*r* = − 0.257, *P* < 0.001), and PLT (*r* = − 0.181, *P* < 0.001) levels were negatively correlated with liver fibrosis, whereas HDL level (*r* = 0.302, *P* < 0.001), fibrosis index based on four factors (FIB-4) (*r* = 0.216, *P* < 0.001), and aspartate transaminase-to-platelet ratio index (APRI) (*r* = 0.188, *P* < 0.001) showed significantly positive correlations with liver fibrosis. The FIB-4 and APRI scores were calculated using formulae from other studies [22, 23].

**Table 3:** Spearman correlation between serum markers and liver fibrosis in patient with *Schistosoma japonicum*

Variables	*r*	*P*
HDL	0.302	< 0.001
LDL	−0.318	< 0.001
HB	−0.257	< 0.001
PLT	−0.181	< 0.001
FIB-4	0.216	< 0.001
APRI	0.188	< 0.001
(HDL × 100)/(LDL × HB)	0.473	< 0.001

*FIB-4* Fibrosis index based on four factors, *APRI* aspartate transaminase-to-platelet ratio index; for other abbreviations, see Tables 1 and 2

### Diagnostic value of individual markers for predicting liver fibrosis in patients infected with *S. japonicum*

**Fig. 1:**
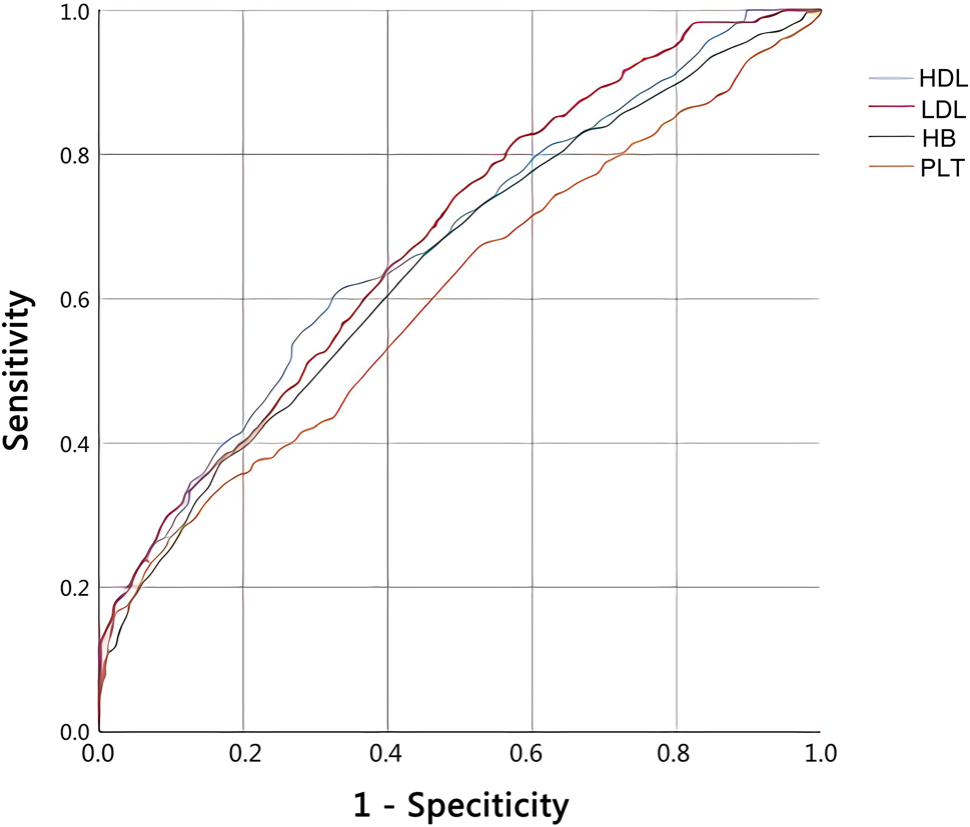
ROC curves of the most statistically significant individual markers to predict liver fibrosis in patient with *Schistosoma japonicum*. *HDL* High-density lipoprotein, *LDL* low-density lipoprotein, *HB* hemoglobin, *PLT* platelets

ROC curves were estimated for all statistically significant individual markers. Table 4 summarizes the estimated AUCs with the corresponding sensitivity, specificity, PPV and NPV for all of the individual markers. The ROC curves for the most statistically significant individual markers are shown in Fig. 1. AUC for HDL was 0.675 (95% CI 0.648–0.701, sensitivity = 60.5%, specificity = 67.4%, *P* < 0.001) with a cut-off value of 1.41 mmol/L. LDL, with a cut-off point of 3.55 mmol/L, showed the highest AUC, 0.683 (95% CI 0.657–0.710, sensitivity = 74.0%, specificity = 50.7%, *P* < 0.001). The AUC of HB was 0.648 (95% CI 0.621–0.676, sensitivity = 66.8%, specificity = 54.3%, *P* < 0.001) with a cut-off value of 134 g/L. PLT had a cut-off point of 131 × 10^9^/L and had the lowest AUC, 0.604 (95% CI 0.575–0.632, sensitivity = 32.7%, specificity = 84.4%, *P* < 0.001).

**Table 4:** Diagnostic value of the different parameters or combined markers for predicting liver fibrosis in patients infected with *Schistosoma japonicum*

	AUC (95% CI)	Cut-off point	Sensitivity (%)	Specificity (%)	PPV (%)	NPV (%)	*P*
HDL	0.675 (0.648–0.701)	1.41	60.5	67.4	65.7	62.2	< 0.001
LDL	0.683 (0.657–0.710)	3.55	74.0	50.7	65.3	60.8	< 0.001
HB	0.648 (0.621–0.676)	134	66.8	54.3	61.2	60.1	< 0.001
PLT	0.604 (0.575–0.632)	131	32.7	84.4	54.8	68.5	< 0.001
FIB-4	0.624 (0.596–0.653)	2.20	46.1	74.3	65.3	57.0	< 0.001
APRI	0.608 (0.580–0.636)	0.49	33.8	83.4	67.8	54.9	< 0.001
(HDL × 100)/(LDL × HB)	0.773 (0.750–0.796)	0.33	62.3	77.4	74.0	66.5	< 0.001

*AUC* Area under receiver operating characteristic curve, *PPV* positive predictive value, *NPV* negative predictive value; for other abbreviations, see Tables 1, 2 and 3

### Establishing a new predictive index for liver fibrosis in patients infected with *S. japonicum*

Based on the Spearman correlation analysis results (the absolute value of *r* is greater than 0.2) and AUCs of individual markers, HDL, LDL and HB were selected to construct a new predictive index, which is expressed by the following formula: (HDL × 100)/(LDL × HB). This new predictive index was positively correlated with liver fibrosis (*r* = 0.473, *P* < 0.001; Table 3), and it had the highest AUC, i.e., 0.773 (95% CI 0.750–0.796, cut-off point = 0.33, sensitivity = 62.3%, specificity = 77.4%, PPV = 74.0%, NPV = 66.5%, *P* < 0.001; Table 4).

### Comparison of the new predictive index with current predictive indexes for liver fibrosis (APRI and FIB-4)

**Fig. 2:**
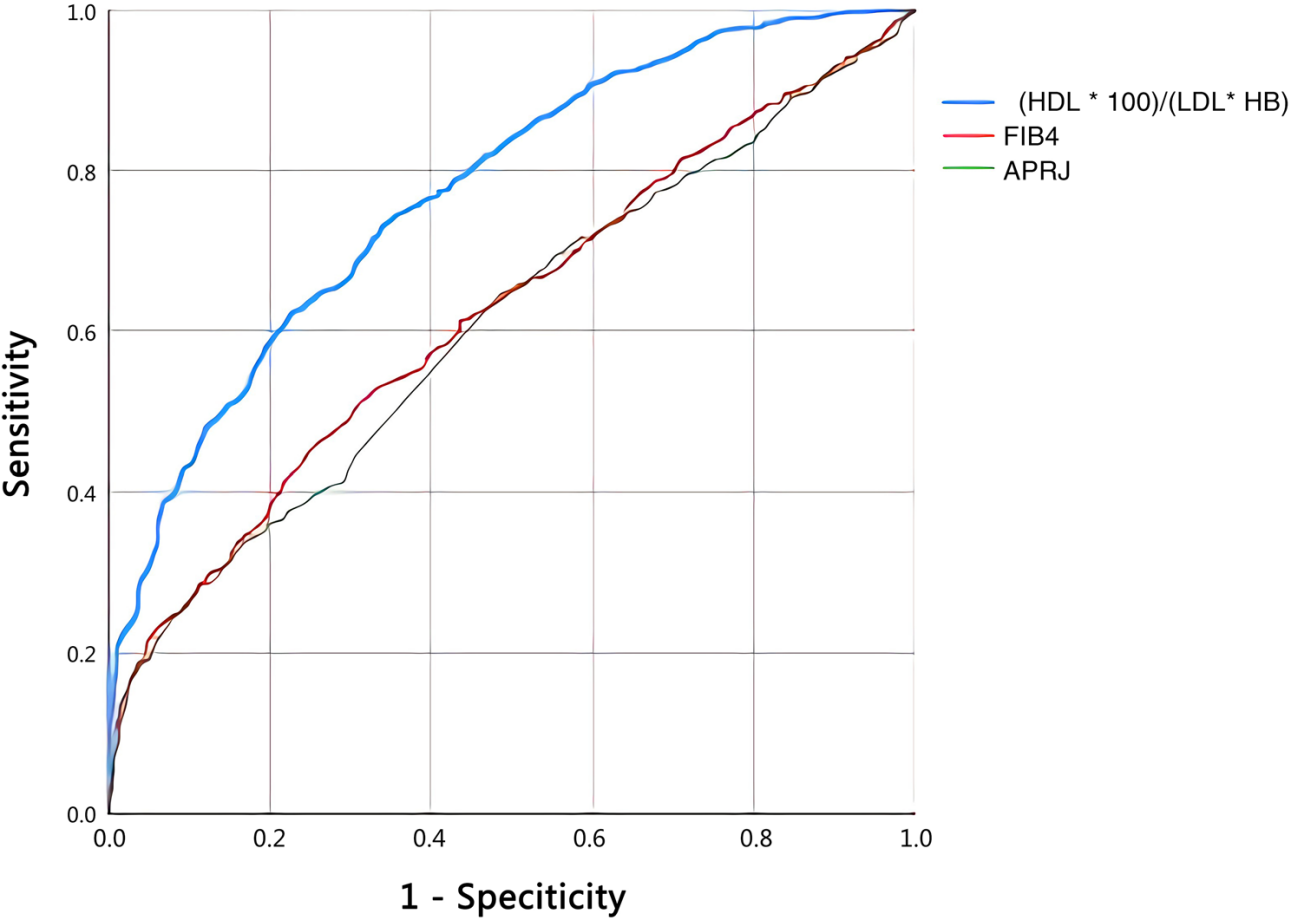
Comparison of the new index with APRI and FIB-4 for ROC curves to predict liver fibrosis in patient with *Schistosoma japonicum*. *HDL* High-density lipoprotein, *LDL* low-density lipoprotein, *HB* hemoglobin, *FIB4* fibrosis index based on four factors, *APRI* aspartate transaminase-to-platelet ratio index

ROC curves were used to evaluate the overall diagnostic performance of the non-invasive models (Fig. 2). As shown in Table 4, the new predictive index (AUC = 0.773, 95% CI 0.750–0.796, sensitivity = 62.3%, specificity = 77.4%, *P* < 0.001) had a better AUC than FIB-4 (AUC = 0.624, 95% CI 0.596–0.653, sensitivity = 46.1%, specificity = 74.3%, *P* < 0.001) and APRI (AUC = 0.608, 95% CI 0.580–0.636, sensitivity = 33.8%, specificity = 83.4%, *P* < 0.001).

## Discussion

In the present study, the main findings were that HDL, LDL, HB and PLT levels were independently associated with liver fibrosis, even after controlling for multiple factors (age, sex, BMI, FBG, AST levels, WBC, RBC, and other lipid profiles). In addition, the new predictive index (combining HDL, LDL and HB) could predict the presence or absence of liver fibrosis in patients infected with *S. japonicum*.

Elevated HDL and reduced LDL levels were independently associated with an increased risk of liver fibrosis in the patients infected with *S. japonicum*. To the best of our knowledge, this is the first study on humans to examine the relationship between lipid profile and liver fibrosis in *S. japonicum*-infected patients. A lipid-fibrosis association has been described in the literature for other liver disease populations, such as patients infected with viral hepatitis, and those with alcoholic and non-alcoholic fatty liver disease. The relationship between HDL levels and liver fibrosis remains inconclusive, with most studies reporting a protective effect of higher HDL with respect to liver fibrosis [16, 24]; the results of only one study are in line with ours, and show that elevated HDL levels are associated with more liver fibrosis [25]. There are several potential explanations for these contradictory results. One possibility is that HDL has a dual nature. Specifically, substances associated with HDL have anti-inflammatory effects in the absence of inflammation, but when inflammation occurs, inflammatory mediators that are transported by HDL have a pro-inflammatory effect [26]. Liver fibrosis is a consequence of severe inflammation [10]. In the process of liver fibrosis, levels of inflammatory mediators increase, thus the level of HDL, which acts as a pro-inflammatory factor involved in the transport of inflammatory mediators [27], also increases. Another possible explanation is that liver fibrosis associated with *S. japonicum* infection may influence the activity of cholesteryl ester transfer protein (CETP) [28], and thus lead to reduced reverse cholesterol transport, which results in lower HDL clearance. In addition, the inconsistent results may be partly explained by effects of *S. japonicum* infection on the lipid profile that have yet to be fully elucidated. Previous studies [29–31] have shown that *S. japonicum* infection can lead to the reprogramming of lipid metabolism, but the specific molecular mechanism of this is complex and needs to be explored in future work.

Similar to previous studies, the LDL levels were inversely associated with liver fibrosis in the patients infected with *S. japonicum*. Valkov et al. [32] reported that a decrease in serum LDL was associated with an increase in advanced fibrosis and cirrhosis in patients infected with chronic hepatitis C. Similar results were shown in another study, which confirmed that lower LDL was an independent predictor of an increase in alcohol-related liver fibrosis [33]. Also, Jaafar et al. [17] showed that lower LDL levels may be a sign of severe liver fibrosis in diabetic patients with nonalcoholic fatty liver disease. These earlier results may be explained by the fact that fibrosis leads to a reduction in the synthesis of apolipoprotein B (ApoB) in the liver [34, 35]. Recent research suggested that phosphatase and tensin homolog deleted on chromosome ten were significantly inhibited in mice infected by *S. japonicum* [29]. Furthermore, another study showed that liver phosphatase and tensin homolog deleted on chromosome ten elimination can reduce ApoB protein mass [36]. Thus, we can speculate that there is also a reduction in the synthesis of ApoB in *S. japonicum* populations, but this needs to be confirmed in future studies on humans.

Moreover, the results of the present study showed that reduced HB and PLT levels were independently associated with liver fibrosis in *S. japonicum*-infected patients. The relationship between routine blood parameters and liver fibrosis in *S. japonicum*-infected patients has been widely studied [37–39]. In agreement with our findings, Wu et al. [39] reported that PLT counts were significantly lower in liver fibrosis patients compared with non-liver fibrosis patients. There are discrepant findings regarding the association between HB and liver fibrosis. The results of Coutinho et al. [40] were consistent with ours indicating that the HB levels of patients with severe fibrosis were lower than those of patients without liver fibrosis; however, Wu et al. [39] found no association between HB levels and liver fibrosis. These inconsistent results may reflect differences in study populations and fibrosis-assessment methods.

Our study generated a new index (HDL × 100/LDL × HB) for predicting liver fibrosis in *S. japonicum*-infected patients. Although the diagnostic value of FIB-4 and APRI for liver fibrosis has been validated for a variety of chronic liver diseases [41–44], our new predictive index had a better AUC than these indices for predicting liver fibrosis in *S. japonicum*-infected patients. This indicates that the diagnostic value of APRI and FIB-4 is too low for the diagnosis of the latter, but this needs to be validated by further prospective studies.

Our data confirm and add to the findings of prior research on the relationship between lipid profile and liver fibrosis in *S. japonicum*-infected patients. However, there are several limitations that need to be addressed. First, this was a retrospective cross-sectional study, so it was not possible to explore a causal relationship between lipid profile and liver fibrosis. Second, ultrasound was used in our study to evaluate liver fibrosis, in accordance with the World Health Organization diagnostic criteria for *S. japonicum*. The accuracy of this technique is lower than that of transient elastography [45] and liver biopsies (the gold standard) [46]. But, due to cost and time constraints, ultrasound is the most widely used clinical diagnostic method for liver fibrosis due to schistosomiasis, and studies have reported that it is suitable for the diagnosis of liver fibrosis due to infection with *S. japonicum* [18, 47]. Third, certain covariates were not included in our study as the relevant data were not available from the patients’ medical records, such as genetic factors, which have been shown to play a crucial role in the development of liver fibrosis [18, 33]. In addition, *Schistosoma* eggs may affect lipid homeostasis [31], and egg burdens may cause an inflammatory response in the liver, leading to liver fibrosis [48]. Therefore, egg counts are also an important covariate that may influence the association between lipids and liver fibrosis. Future research should include these factors to further confirm that lipid profile is independently associated with liver fibrosis. Finally, the new predictive index generated during this study showed moderate sensitivity for the identification of liver fibrosis in patients infected with *S. japonicum*.

## Conclusions

In summary, HDL, LDL, HB and PLT levels were independently associated with liver fibrosis in patients infected with *S. japonicum*. Moreover, the new predictive index presented here (HDL × 100/LDL × HB) can predict the presence or absence of liver fibrosis in *S. japonicum*-infected patients. This index can help clinicians to more easily determine liver fibrosis and to develop effective treatment and follow-up strategies for *S. japonicum*-infected patients. Due to the potential complexity and severity of *S. japonicum* infections and liver fibrosis, it is important to further explore their underlying molecular mechanisms.

## Data Availability

The datasets used and/or analyzed during the current study are available from the corresponding author upon reasonable request.

## Abbreviations

*S. japonicum*: *Schistosoma japonicum*
ALT: Alanine aminotransferase
APRI: Aspartate transaminase-to-platelet ratio index
AST: Aspartate aminotransferase
AUC: Area under receiver operating characteristic curve
BMI: Body mass index
CI: Confidence interval
FBG: Fasting blood glucose
FIB-4: Fibrosis index based on four factors
HB: Hemoglobin
HDL: High-density lipoprotein
LDL: Low-density lipoprotein
OR: Odds ratio
PLT: Platelets
RBC: Red blood cell count
ROC: Receiver operating characteristic
TC: Total cholesterol
TG: Triglyceride
WBC: White blood cell count
