# Correction: Association between serum lipid profile and liver fibrosis in patients infected with *Schistosoma japonicum*

**DOI:** 10.1186/s13071-022-05470-w

**Published:** 2022-09-19

**Authors:** Yang Liu, PengPeng Zhang, JunHui Li, Hao Li, Chen Zhou, Yu Zhang, YingZi Ming

**Affiliations:** 1 Transplantation Center, Third Xiangya Hospital, Central South University, Changsha, China; 2 Engineering and Technology Research Center for Transplantation Medicine, National Health Commission, Changsha, 410013 China

## Correction to: Parasites & Vectors (2022) 15:268 10.1186/s13071-022-05359-8

Following publication of the original article [1], the authors flagged an error in the legend of Fig. 2: 'APRJ' had been written in place of 'APRI'. The caption has since been corrected in the published article. The authors thank you for reading and apologize for any inconvenience caused.

## References

[1] Liu Y, Zhang P, Li J, Li H, Zhou C, Zhang Y, Ming Y (2022). Association between serum lipid profile and liver fibrosis in patients infected with *Schistosoma japonicum*. Parasit Vectors.

